# Variable Selection for Sparse Data with Applications to Vaginal Microbiome and Gene Expression Data

**DOI:** 10.3390/genes14020403

**Published:** 2023-02-03

**Authors:** Niloufar Dousti Mousavi, Jie Yang, Hani Aldirawi

**Affiliations:** 1 Department of Mathematics, Statistics, and Computer Science, University of Illinois at Chicago, Chicago, IL 60607, USA; 2 Department of Mathematics, California State University—San Bernardino, San Bernardino, CA 92407, USA

**Keywords:** zero-inflated model, hurdle model, longitudinal data, model selection, vaginal microbiome, gene expression

## Abstract

Sparse data with a high portion of zeros arise in various disciplines. Modeling sparse high-dimensional data is a challenging and growing research area. In this paper, we provide statistical methods and tools for analyzing sparse data in a fairly general and complex context. We utilize two real scientific applications as illustrations, including a longitudinal vaginal microbiome data and a high dimensional gene expression data. We recommend zero-inflated model selections and significance tests to identify the time intervals when the pregnant and non-pregnant groups of women are significantly different in terms of *Lactobacillus* species. We apply the same techniques to select the best 50 genes out of 2426 sparse gene expression data. The classification based on our selected genes achieves 100% prediction accuracy. Furthermore, the first four principal components based on the selected genes can explain as high as 83% of the model variability.

## 1. Introduction

Sparse or zero-inflated data has a lot of applications in various disciplines such as microbiome [1], gene expression [2], epidemiology [3], health care [4], security [5], social networks [6], and more. Modeling sparse data is very challenging due to the high proportion of zeros and severe skewness of the distribution [7,8]. Modeling sparse data appropriately is also critical for successful scientific applications. For example, zero readings in the microbiome and RNA-seq data have two possible sources: First, some species or genes exist but are not detected as a result of insufficient sequence depth or inefficiencies of the technology processes (non-biological zeros); secondly, it is possible that some species or genes are truly never represented (biological zeros) [9].

To model sparse data, zero-inflated and hurdle models have been widely used. Both of them consist of two data-generating processes. The first process generates purely zeros, while the second one is governed by some distribution, for example, Poisson distribution, which may or may not generate zeros. The zero-inflated Poisson (ZIP) model was proposed by Lambert (1992) with an application to defects in manufacturing [10]. In 1994, Greene considered the zero-inflated negative binomial (ZINB) model with an application on consumer behavior and default on credit card loans [11].

In this paper, we consider analyzing zero-inflated data in a more general and complex context. One motivating example is the vaginal microbiome data, which is longitudinal, and the goal is to identify the time intervals when the two groups of individuals are significantly different [2].

The vaginal microbiome is a dynamic micro-ecosystem that inhabits the vaginal surfaces and its cavity. The vaginal microbiome has great significance in maintaining vaginal health and protecting the host from urogenital diseases such as sexually transmitted diseases [12]. Normal flora appears dominated by one or two species of *Lactobacillus*. *Lactobacilli* are the most abundant vaginal bacteria in women [13]. It is known that 92% of the normal vaginal flora consists of *Lactobacillus* species [14]. *Lactobacilli* produce lactic acid, which acidifies the vagina to pH <4 to restrict the growth of all bacteria and protect the vagina against pathogens. *Lactobacilli* also produce hydrogen peroxide (H${}_{2}$O${}_{2}$) to kill bacteria cells by destroying their cell walls [15]. Several bacteriocins produced by different *Lactobacillus* species have been described [16,17]. The absence of vaginal *Lactobacilli* has a negative effect on women’s health. For example, vaginal *Lactobacilli* have an impact on In Vitro Fertilization (IVF) success rate. A very recent study shows that women with a low percentage of *Lactobacillus* in the vaginal microbiome have a lower rate of success in embryo implantation and embryo transfer [18].

Modeling *Lactobacillus* species is a growing research area. For example, Romero et al.  (2014) proposed a longitudinal study for comparing the vaginal microbiome between pregnant and non-pregnant women [2]. The mixed effects modeling of the reads count data on the pregnancy status was performed using the zero-inflated negative binomial mixed-effects (ZINBLME) models. In addition, negative binomial linear mixed effects (NBLME) and Poisson linear mixed effects (PLME) models were used in a comparison. The ZINBLME model provided the optimal fit based on AIC values. Chen and Li (2016) proposed a zero-inflated beta regression model with random effects (ZIBR) for testing the association between *Lactobacillus* species and clinical covariates for a longitudinal vaginal microbiome study [19]. Both simulation studies and the real application data have shown that the ZIBR model outperformed the previously used methods such as zero-inflated Poisson, binomial, and negative binomial regression models with random effects. Zhang et al. (2020) proposed zero-inflated Gaussian mixed models (ZIGMMs) to analyze longitudinal vaginal microbiome data [20]. The Expectation-Maximization (EM) algorithm was used to fit the ZIGMMs. The study concluded that ZIGMMs is a robust and flexible method compared to some other models such as linear mixed models (LMMs), negative binomial mixed models (NBMMs), and zero-inflated beta regression mixed models (ZIBR).

Another motivating example is the gene expression data [21], which involves 2426 genes and is high-dimensional. The goal is to select a small group of genes for labeling five categories.

Over the past few decades, RNA sequencing (RNA-Seq) has been frequently used in genomics, biological, medical, and drug research. Poisson, negative binomial, zero-inflated Poisson (ZIP), zero-inflated negative binomial (ZINB), and Bayesian methods have been widely used to model a single cell RNA-seq data [22,23,24,25]. For example, McDavid et al. (2013) proposed a chi-square asymptotic distribution of the likelihood ratio test to compute *p* values and assess the significance [26]. They showed that their test is more powerful than *t*-test on zero-inflated data. Kharchenko et al. (2014) proposed a Bayesian model for single-cell differential expression analysis [25]. They found out that their proposed method has a higher sensitivity than commonly used RNA-seq differential expression methods (DESeq and CuffDiff) and the zero-inflated approach developed by McDavid et al. (2013) [26]. Zero-inflated beta regression (ZIBSeq) approach was developed by Peng et al. (2016) for identifying differential abundant metagenomics features between multiple clinical conditions [27]. Compared with other available methods, the ZIBSeq approach demonstrates better performance with larger AUC (area under the curve) values for human metagenomics data and some simulation studies.

To analyze sparse data, there are some R packages available from the Comprehensive R Archive Network (CRAN, https://cran.r-project.org/, accessed on 27 October 2022), including “bzinb” [28], “hurdlr” [29], “iZID” [30], “gamlss” [31], “pscl” [32], “mhurdle” [33], “rbtt” [34], “ZIBseq” [35], “zic” [36], “ZIM” [37], “ziphsmm” [38], etc. Among them, iZID covers 12 different discrete distributions including Poisson, negative binomial, beta binomial, beta negative binomial, and their zero-inflated and hurdle versions [39]. It implemented the bootstrapped Monte Carlo *p* value estimates for identifying a discrete distribution [7]. In this paper, we recommend a newly developed R package, “AZIAD” [40], which covers 27 discrete and continuous distributions and resolves some limitations of the other R packages [41]. The AZIAD package provides maximum likelihood estimates (MLE) for model parameters, Kolmogorov-Smirnov tests (KS test) for fairly general distributions, likelihood ratio tests (LRT) for model selection, Fisher information matrix and confidence intervals for parameter estimates. We provide more details on utilizing AZIAD in Section 2.4.

In this paper, we use the two motivating examples as illustrations on analyzing longitudinal or high-dimensional sparse data. For the longitudinal vaginal microbiome data, we compare the pregnant and non-pregnant groups in terms of the *Lactobacillus* species to identify the time intervals when the two groups are significantly different. Although we use the *Lactobacillus* species as an example, our methods and analysis can be applied to any other vaginal microbiome species in the dataset as well. For the gene expression data, we select the most informative genes based on sparse data model selection and show how the selected genes help with predicting the class labels. We also apply the principal component analysis to the top 50 selected genes and show that the four principal components can explain 83% of the model variability.

## 2. Materials and Methods

To analyze sparse data, we use zero-altered models (or hurdle models) and zero-inflated models. In this section, we first briefly review zero-inflated and hurdle models. Then we illustrate by examples how to use AZIAD for selecting the most appropriate model for sparse data. One of our major contributions is the significance test that we develop based on sparse data model selection, which will be used for selecting significant time points for longitudinal sparse data and selecting the most informative covariates for high-dimensional sparse data.

### 2.1. Zero-Altered or Hurdle Models

Zero-altered models, also known as *hurdle models*, have been widely used for modeling sparse data (see, for example, [1] or [8], for a good review). Technically speaking, hurdle models can also be used for modeling data with a number of zeros less than expected. A general hurdle model consists of two components, one generating zeros and the other generating non-zeros. Given a baseline distribution function $f_{\theta }\left(y\right)$, where the parameter(s) $\theta ={\left(\theta_{1},\ldots ,\theta_{p}\right)}^{T}$, p≥1, the distribution function of the corresponding hurdle model can be written as follows

(1) $$f_{\mathrm{ZA}}\left(y|\phi ,\theta \right)=\phi 1_{\left\{y=0\right\}}+\frac{1-\phi }{1-p_{0}\left(\theta \right)}f_{\theta }\left(y\right)1_{\left\{y\neq 0\right\}}$$

where ϕ∈[0,1] is the weight parameter of zeros, $f_{\theta }\left(y\right)$ is either a probability mass function (pmf) for some discrete baseline distribution or a probability density function (pdf) for some continuous baseline distribution, and $p_{0}\left(\theta \right)=f_{\theta }\left(0\right)$ for discrete distributions or simply 0 for continuous distributions.

### 2.2. Zero-Inflated Models

Unlike zero-altered models, a zero-inflated model always assumes an excess of zeros. Besides zeros coming from the baseline distribution $f_{\theta }\left(y\right)$, there are additional zeros modeled by a weight parameter ϕ∈[0,1]. The distribution function of the zero-inflated model can be defined as follows

(2) $$f_{\mathrm{ZI}}\left(y|\phi ,\theta \right)=\left[\phi +\left(1-\phi \right)p_{0}\left(\theta \right)\right]1_{\left\{y=0\right\}}+\left(1-\phi \right)f_{\theta }\left(y\right)1_{\left\{y\neq 0\right\}}$$

### 2.3. Zero-Altered and Zero-Inflated Models with Continuous Baseline Distributions

When the baseline distribution $f_{\theta }\left(y\right)$ is continuous, $p_{0}\left(\theta \right)=0$ and models (1) and (2) are the same, called a *zero-altered and zero-inflated* (ZAZI) model (see [41] for more details). Its distribution function can be written as follows

(3) $$f_{\mathrm{ZAZI}}\left(y|\phi ,\theta \right)=\phi 1_{\left\{y=0\right\}}+\left(1-\phi \right)f_{\theta }\left(y\right)1_{\left\{y\neq 0\right\}}$$

Commonly used continuous baseline distributions include Gaussian (or normal), log-normal, half-normal, exponential, etc. The corresponding zero-inflated models are also known as zero-inflated Gaussian (ZIG), zero-inflated log-normal (ZILN), zero-inflated half-normal (ZIHN), and zero-inflated exponential (ZIE), respectively.

### 2.4. Model Selection Using AZIAD Package

To identify the most appropriate model for sparse data, we recommend the R package AZIAD. Compared with other existing R packages on analyzing zero-inflated data, (1) it takes 27 different distributions under consideration; (2) it covers both discrete and continuous baseline distributions; (3) it provides Fisher information matrix and confidence intervals for estimated parameters as well.

When the baseline distribution is continuous with the pdf $f_{\theta }\left(y\right)$, AZIAD covers normal, log-normal, half-normal, exponential, and their corresponding zero-inflated and hurdle models. When the baseline distribution is discrete with the pmf $f_{\theta }\left(y\right)$, the package covers Poisson, geometric, negative binomial, beta binomial, beta negative binomial, and their corresponding zero-inflated and hurdle models.

We apply KS-test for each model under our consideration to test if the model is appropriate for the given sparse data. If the *p* value of the KS-test is below 0.05, we usually discard the corresponding model. There are two functions built in the AZIAD package, kstest.A and kstest.B. According to [41], kstest.B is recommended for data with a sample size of about 50 or below, such as the vaginal microbiome data of the pregnant group on week three (see Section 3.1), while kstest.A is recommended for a larger sample size, such as the gene expression data (see Section 3.2). We provide below two toy examples.

> set.seed(456)

> Data1=sample.h1(2000,phi=0.3,dist="normal",mean=10,sigma=2)

> kstest.A(Data1,nsim=100,bootstrap=TRUE,dist="normalh",

lowerbound=1e-10,upperbound=100000)$pvalue

> 1

> kstest.A(Data1,nsim=100,bootstrap=TRUE,dist="lognormal",

lowerbound=1e-1,upperbound=1000000)$pvalue

>  0

> kstest.A(Data1,nsim=100,bootstrap=TRUE,dist="zilognorm",

lowerbound=1e-1,upperbound=1000000)$pvalue

> 0

> Data2=sample.zi1(N=30,phi=0.4,r=10,alpha1=3,alpha2=5,dist="bnb")

> kstest.B(Data2,nsim=100,bootstrap=TRUE,dist="zibnb",

lowerbound=1e-10,upperbound=100000)$pvalue

> 0.76

> kstest.B(Data2,nsim=100,bootstrap=TRUE,dist="zip",

lowerbound=1e-10,upperbound=100000)$pvalue

> 0

The R function sample.h1 can be used for generating random samples from hurdle models. We first generate a random sample (Data1) from a normal hurdle distribution with parameters (ϕ,μ,σ)=(0.3,10,2) and sample size N=2000. For reproducibility purposes, we set a random seed 456. For this data, we apply kstest.A to three different distributions, normal hurdle, log-normal, and zero-inflated log-normal. The results show that only the true distribution normal hurdle is appropriate with a *p* value larger than 0.05.

We then generate a random sample Data2 from a zero-inflated beta negative binomial (ZIBNB) model using the R function sample.zi1. The model parameters are (ϕ,r,α,β)=(0.4,10,3,2) and the sample size is N=30. Since the sample size is below 50, we apply kstest.A to two models, ZIBNB and ZIP. Again only the true model ZIBNB has a *p*-value larger than 0.05.

To develop our significance test (see Section 2.5) for variable selection, we also need to calculate the maximum likelihood estimate (MLE), which maximizes the likelihood function, for each model under consideration. We use the AZIAD built-in R functions, new.mle for general baseline distributions, and zih.mle for zero-inflated and hurdle models. To demonstrate in more detail, we consider the toy examples as follows.

> library(AZIAD)

> set.seed(657)

> Data1=extraDistr::rbbinom(1000,size=4,alpha=2,beta=3)

> new.mle(Data1,n=10,alpha1=3,alpha2=4,dist="bb")

>     n    Alpha     Beta    loglik

>   3.99 1.975527 2.923279 -3060.583

> Data2=sample.zi1(2000,phi=0.3,dist=’bnb’,r=5,alpha=3,alpha2=3)

> zih.mle(Data2,r=10,alpha1=3,alpha2=4,dist="bnb.zihmle",type="zi")

>        r   alpha1   alpha2       phi    loglik

>   5.095388 3.033706 2.902682 0.3025823 -5091.443

> Data3=sample.h1(2000,phi=0.3,dist="lognormal",mean=1,sigma=4)

> zih.mle(Data3,mean=4,sigma=2,dist="lognorm.zihmle",type="h")

>     mean    sigma    phi    loglik

>    1.049724 3.931015 0.3095 -6537.076

> Data4=sample.zi1(2000,phi=0.3,dist="exponential",lambda=20)

> zih.mle(Data4,lambda=10,dist="exp.zihmle",type="zi")

>    lambda   phi  loglik

>    19.55911 0.305   1513

For illustration purposes, the data sets are simulated from beta binomial (BB) with true parameters (n,α,β)=(4,2,3), ZIBNB with true parameters (ϕ,r,α,β)=(0.3,5,3,3), log-normal hurdle with true parameters (ϕ,μ,σ)=(0.3,1,4), and ZIE with true parameters (ϕ,λ)=(0.3,20), respectively. As observed, our corresponding estimates $\left(\hat{n},\hat{\alpha },\hat{\beta }\right)=\left(3.99,1.97,2.92\right)$, $\left(\hat{\phi },\hat{r},\hat{\alpha },\hat{\beta }\right)=\left(0.3,5.09,3.03,2.90\right)$, $\left(\hat{\phi },\hat{\mu },\hat{\sigma }\right)=\left(0.3,1.04,3.93\right)$, and $\left(\hat{\phi },\hat{\lambda }\right)=\left(0.3,19.55\right)$ are reasonably close to the true parameter values. Note that the initial values of the parameters are required but fairly flexible.

### 2.5. Significance Test on Group Labels

In this section, we propose a significance test for selecting the most informative covariates associated with group labels of sparse data.

First of all, we briefly review two commonly used model selection criteria, Akaike information criterion (AIC) and Bayesian information criterion (BIC), defined as

$$\begin{array}{ccc} AIC & = & -2\cdot loglik+2\cdot \;k\\ BIC & = & -2\cdot loglik+(logN)\cdot k \end{array}$$

where loglik is the maximized likelihood (see Section 2.4), *k* stands for the number of parameters in the model, and *N* is the sample size (see, for example, [42] for more details). According to Hastie et al. (2009), BIC is asymptotically consistent in the sense that it will choose the correct model with a probability approaching to 1 as the sample size N→∞, and AIC is more suitable for small or moderate sample sizes [42].

In this paper, we adopt AIC since the sample size is 52 for the vaginal microbiome data (see Section 3.1), or 801 for the gene expression data (see Section 3.2).

Now we propose a significance test based on our sparse data model selection procedure. In general, we consider a data set with covariates $\left\{x_{ij}|i=1,\ldots ,N;j=1,\ldots ,p\right\}$, that is, consisting of *N* individuals and *p* covariates, and class labels $y_{i}\in \left\{1,\ldots ,m\right\}$, m≥2. The goal is to select the most informative covariates for predicting the class labels. For each covariate, say the *j*th covariate, the readings or measures $x_{ij},i=1,\ldots ,N$ are grouped into *m* blocks according to their class labels $y_{i},i=1,\ldots ,N$. We summarize the following procedure in three steps.

- **Step 1**: Choose the most appropriate model for all the *N* numbers, $\left\{x_{ij}|i=1,\ldots ,N\right\}$ after ignoring their class labels. This task is accomplished by performing KS-tests using kstest.A on all models under consideration (see also [7]). Then we compute the MLE of the parameters for the chosen model using R function zih.mle. The corresponding AIC value is denoted by $ModelI_{AIC}$.
- **Step 2**: For each of the *m* classes, say the *k*th class, we choose the most appropriate model for the data $\left\{x_{ij}|y_{i}=k\right\}$ of the *k*th class, compute the MLE and denote the corresponding AIC value by AIC(k). Then aggregated AIC value $ModelII_{AIC}$ is essentially the summation of the AIC values from *m* classes, that is, $ModelII_{AIC}=\sum_{k=1}^{m}AIC\left(k\right)$.
- **Step 3**: Take the difference of two AIC values with or without class labels, $ModelI_{AIC}-ModelII_{AIC}=ModelI_{AIC}-\sum_{k=1}^{m}AIC\left(k\right)$. A larger difference indicates that the *j*th covariate is more informative for predicting the class labels.

We refer the readers to [43] for more discussion on using AIC or BIC for model selection.

## 3. Two Applications

In this section, we use two real examples to illustrate how our variable selection techniques could be used for sparse data analysis.

### 3.1. Vaginal Microbiome

The purpose of this study is to characterize the changes in the composition of the vaginal microbiome (including *Lactobacilli*) at some time points between two groups of women, pregnant or non-pregnant. The dataset is available in Romero et al. (2014)’s study [2]. The original study includes 32 non-pregnant women and 22 pregnant women who had a term delivery without complications. Non-pregnant women self-sampled with a frequency of twice a week for 16 weeks. Pregnant women had a speculum examination at each visit when a sample of vaginal fluid was collected as well. Samples were collected every 4 weeks till week 24 of pregnancy and every 2 weeks till delivery. The numbers of samples for the 22 pregnant women are not balanced and fluctuate between 3 and 8. In our analysis, we remove one sample of non-pregnant women since there was only one observation. That is, 31 non-pregnant women are kept for our analysis.

The goal of our study in this paper is to observe the AIC differences between the pregnant and non-pregnant women over the time of pregnancy (1≤t≤38) concentrated on the *Lactobacillus* microbiome and identify time intervals when the two groups of women are significantly different in terms of *Lactobacillus*. Eventually, we can extend this procedure and do parallel analysis for any other species in this dataset.

### 3.2. RNA-Seq Gene Expression Data

The RNA-seq gene expression dataset is a high-dimensional dataset consisting of 801 tissue samples (N=801) and 20,531 genes (*p* = 20,531) [21]. The 801 samples are labeled by 5 different types of cancerous tumors, BRCA, KIRC, COAD, LUAD, and PRAD. The data can be accessed from the UCI Machine Learning Repository (https://archive.ics.uci.edu/ml/datasets/gene+expression+cancer+RNA-Seq#, accessed on 4 November 2022) and were collected as a part of the Cancer Genome Atlas (TCGA) analysis project [44] (see also [21]).

The goal of our study is to select the most informative genes for identifying class labels.

## 4. Data Analysis and Results

In this section, we explain the data analysis procedures and report our results for the two real applications described in Section 3.

### 4.1. Vaginal Microbiome

There are two challenging aspects of this dataset, missing time points and unbalanced time intervals. Our first step is data imputation for the missing time points using *k*-nearest neighbor, where k=1 is used in our study (see, for example, [45] for other possible missing value imputation strategies). Next, we apply linear interpolations on the readings of each individual using R function approx with option method="linear". We select 38 discrete, equally distanced time points on the curves. At each t=1…38, we gather 53 values of *Lactobacillus* microbiome which belongs to the two groups of women. By screening all samples, we find out that all of the samples contain approximately 5% to 13% of zeroes. Therefore, zero-inflated models are more appropriate in model selection.

**Figure 1:**
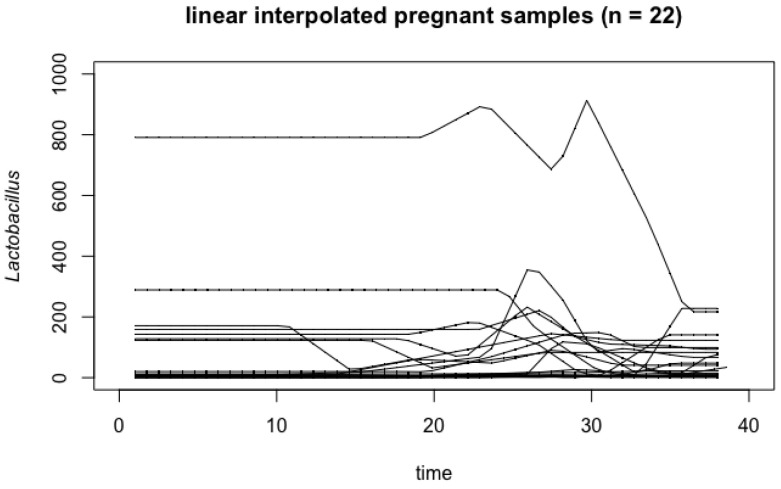
Linear interpolated *Lactobacillus* readings of 22 pregnant women over 38 weeks of pregnancy.

**Figure 2:**
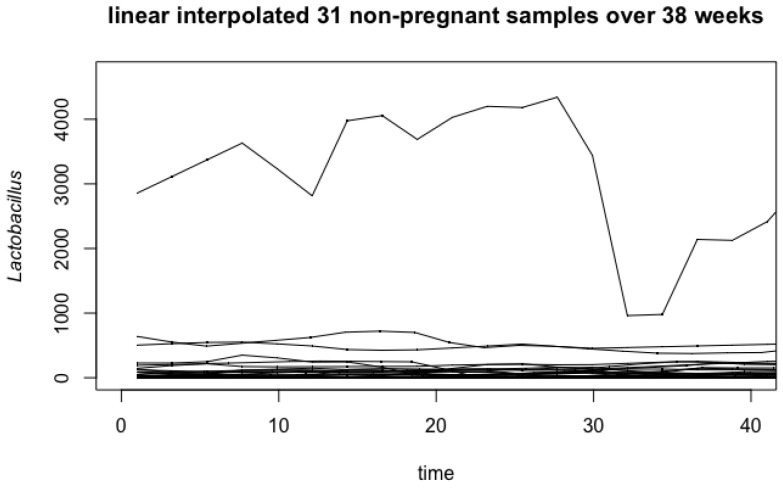
Linear interpolated *Lactobacillus* readings of 31 non-pregnant women over 38 weeks.

Figure 1 and Figure 2 summarize the result of 22 linear interpolated curves for pregnant women, and 31 linear interpolated curves for non-pregnant women over the time of pregnancy (38 weeks). As observed in the non-pregnant samples (Figure 2), there is an apparent outlier curve that acts very differently from the other samples. We remove this outlier since it is influential for our analysis (see Appendix A for more information). As a result, our analysis is based on 30 non-pregnant women and 22 pregnant women.

After the data preparation, we perform the significance test described in Section 2.5. Since the readings are real numbers, we consider 12 models including four continuous distributions, normal (N), half-normal (HN), log-normal (LN), exponential (E), and their zero-inflated and hurdle versions. Based on the KS-test *p* values (see Table 1), we obtain several candidate models with *p* values larger than 0.05. Among them, normal (N) and normal hurdle (NH) are the best ones (for readers’ reference, an extended version of Table 1 can be found in the Supplementary Materials). We choose NH instead of N since the data contains a proportion of zeros. We then calculate $ModelI_{AIC}$ for each of the 38 time points.

As Step 2, we apply the same procedure to pregnant and non-pregnant groups separately. The proportions of zeros in the non-pregnant group are roughly between 10% and 20%, which implies that sparse model should be used. The proportions of zeros in the pregnant group are less than the non-pregnant group’s. Some samples contain 4% of zeroes at the beginning of the pregnancy, while no sample has any zeroes after week 21. Therefore, we consider sparse models for weeks 1–21, and regular continuous models on weeks 22–38. Based on the KS-tests on the non-pregnant group (see Table A1 in Appendix B), we choose the normal hurdle (NH) model again. For the pregnant group, NH model is chosen for weeks 1–21, and normal distribution is chosen for weeks 22–38 (see Table A2 in Appendix B). In this case,

$$ModelII_{AIC}=AIC_{non-pregnant(n=30)}+AIC_{Pregnant(n=22)}$$

**Figure 3:**
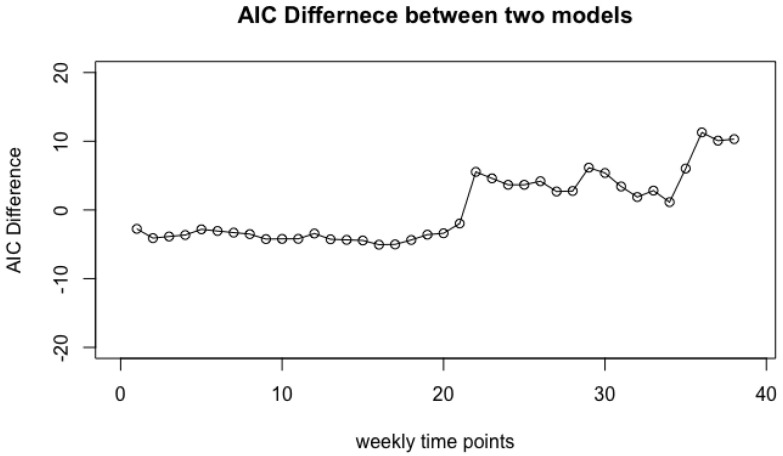
AIC difference $ModelI_{AIC}$−$ModelII_{AIC}$ over 38 weeks of pregnancy.

As Step 3, we calculate $ModelI_{AIC}-ModelII_{AIC}$ for each t=1,…,38. Figure 3 demonstrates the difference of AIC values over the weeks of pregnancy 1≤t≤38 in terms of *Lactobacillus* microbiome. It indicates that the two groups tend to be significantly different after week 22 (AIC differences are larger than 2). It means that the pregnant women and non-pregnant women are significantly different during weeks 22–38 in terms of *Lactobacillus* species.

**Figure 4:**
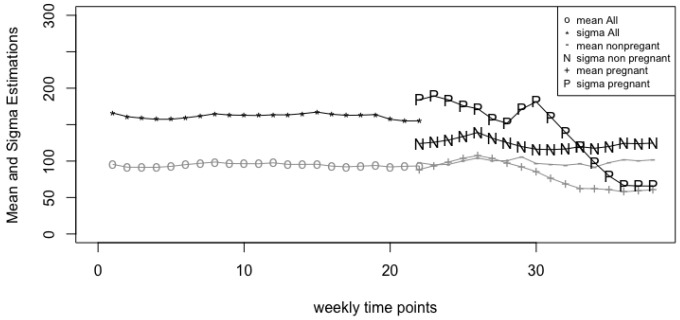
Change of parameter estimates $\hat{\mu }$, $\hat{\sigma }$ over 38 weeks.

**Figure 5:**
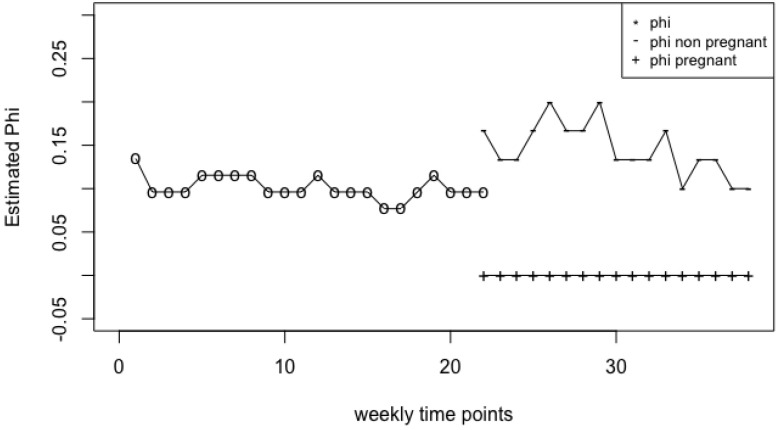
Change of $\hat{\phi }$ estimate over 38 weeks.

To further investigate the differences between the two groups before and after week 22 of pregnancy observed in Figure 3, we conduct a more detailed analysis on the estimated parameters (i.e., $\left(\hat{\mu },\hat{\sigma },\hat{\phi }\right)$) over time. Figure 4 and Figure 5 demonstrate the changes of individual parameters over time. Before week 22, we consider the changes of the estimated parameters for combined groups (N=52), while after week 22 we consider the changes of estimated parameters for the two groups separately. Figure 4 shows that the estimated means of the pregnant group is not so different from the non-pregnant group until week 28. Nevertheless, it is clear that the mean differences are fairly large at the end of the pregnancy. On the other hand, the estimated variances are quite different between the two groups right after week 21. The estimated variance in the pregnant group seems to be much larger than the non-pregnant group’s from weeks 22 to 32, and then becomes smaller than the non-pregnant group’s especially at the end of the pregnancy. The down difference is also significant at the end of the pregnancy. This phenomenon indicates that *Lactobacillus* species is significantly less in the pregnant group compared to the non-pregnant group at the end of the pregnancy. These conclusions are free of the proportions of zeros in the data.

Figure 5 shows the changes of the weight parameter ϕ of zeros over time. The parameter estimates are different between the two groups after week 22, which might be due to the missing counts in the non-pregnant group. The estimated ϕ is 0 after week 22 in the pregnant group, which might be because the pregnant group does examinations regularly, especially when it is close to the end of the pregnancy.

In the microbiome literature, there are some other available methods for analyzing longitudinal microbiome data. For example, MetaLonDA is an R package capable of identifying significant time intervals of microbiome features [46]. It relies on two modeling components, the negative binomial distribution assumption for modeling the read counts, and the semi-parametric SSANOVA technique for modeling longitudinal profiles associated with different phenotypes. For comparison purposes, we apply the MetaLonDA package to the *Lactobacillus* species and compare the results with ours. The below MetaLonDA interval *p* value results (weeks 2–38) claim that there is no significant difference between the two groups during the first 11 weeks and during weeks 22–31, while the differences are significant in weeks 12–21 and after week 31.

$intervals.pvalue

[1] 1.00000 1.00000 1.00000 1.00000 1.00000 1.00000 1.00000 1.00000

[9] 1.00000 1.00000 0.00000 0.00000 0.00000 0.00000 0.00000 0.00000

[17] 0.00000 0.00000 0.00000 0.00000 1.00000 1.00000 1.00000 1.00000

[25] 1.00000 1.00000 1.00000 1.00000 1.00000 0.69333 0.00000 0.00000

[33] 0.00000 0.00000 0.00000 0.00000 0.00000

Our analysis result matches the MetaLonDA result in some time intervals and is different in a small number of time intervals. The differences might be due to two reasons. First, the data mainly consists of real numbers while the negative binomial distribution assumed by MetaLonDA is for integers (counts). Secondly, the data contains a good proportion of zeros, which indicates zero-inflated or hurdle models may fit the data better.

For example, according to MetaLonDA, there is no significant difference between the two groups from week 22 to week 31, which is different from our result. From Figure 4 and Figure 5, one can see that the standard deviations ($\hat{\sigma }$) and the proportions of zeroes ($\hat{\phi }$) are quite different between the two groups starting week 22, although the group means ($\hat{\mu }$) are fairly close. MetaLonDA is not able to detect such differences. For readers’ reference, we provide the estimated parameters of the non-pregnant and pregnant groups starting week 21 in Table S4 of the Supplementary Materials.

### 4.2. RNA-Seq Gene Expression Data

The RNA-seq gene expression data is a high-dimensional dataset consisting of 801 samples and 20,531 genes (covariates). At our data preparation and screening stage, we first delete 267 genes (columns) which contain only zeros. Secondly, we remove the columns (12,356 genes) that have no zeros at all so that we can focus on zero-inflated covariates (covariates without zeros are not sparse and can be dealt with classical data analysis). For the same reason, we choose only the genes (columns) that carry a good proportion (from 5% to 50%) of zeros. Therefore, the total number of genes used for our study is N=2426, which is still high-dimensional.

The 801 gene expression samples are classified into five cancer categories: BRCA, KIRC, COAD, LUAD, and PRAD, which contain 300, 146, 78, 141, and 136 samples, respectively. We use R package AZIAD for the rest analysis. Since the data consists of non-negative real numbers, we consider the same set of 12 models as in Section 4.1. For each of the 2426 genes, we apply the three steps as described in Section 2.5. As Step 1, we apply R function kstest.A to 801 gene expression levels of the gene under consideration after ignoring the labels. Technically speaking, any model with a KS-test *p* value larger than 0.05 could be a candidate. In this study, we choose the model with the largest *p* value for simplicity. In case of ties, we always choose the last one in the tie list.

As a summary of the model selection results for all 801 samples, among the 2426 genes, normal hurdle (NH) model is chosen for 1,194 genes, log-normal hurdle (LNH) model is chosen for 885 genes, log-normal (LN) model is chosen for 98 genes, and zero-inflated exponential (ZIE) model is chosen for another 98 genes. The rest genes are fitted with exponential (66 genes), normal (60 genes), zero-inflated half-normal (22 genes), and half-normal (3 genes) models. We also perform model selections for each of the five categories. The results are slightly different. Log-normal hurdle model is in favor since it is either comparable or better than normal hurdle model in each category. For example, for COAD category, log-normal hurdle model passes 99% of KS-tests compared with 97% of normal hurdle model. After finishing the three steps for each gene (see Section 2.5), we are able to rank the 2426 genes based on their AIC differences from the largest one to the smallest one.

A critical question is how many genes should be chosen for predicting the class labels. To choose a good threshold, we utilize the 1-nearest neighbor classifier (see, for example, [42]) with a various number of selected genes to predict the class labels. Table 2 lists the predicted error rate (also known as the training error rate since all 801 class labels have been used by the classifier) by 1-nearest neighbor classifier with 20, 50, 100, or 2426 ranked genes. For comparison purpose, we also the prediction error rate (0.02) of 1-nearest neighbor classifier based on the top 7 principle components suggested by [21]. Note that the principle components here are based on 20,264 genes after removing the first column (sample indices) and all zero columns. According to Table 2, 50 seems to be a good option for the number of selected genes, whose prediction error (0.0037) is just below the top 7 PCA’s. For readers’ reference, we provide the list of the top 50 genes in Appendix C.

It is known that the training error rate may underestimate the true prediction error rate due to over-fitting [42]. To obtain a fair estimate for the prediction error, we conduct a 5-fold cross-validation on this data. More specifically, we split the data (N=801) into 5 roughly equal-sized blocks, with each containing about 160 samples. For each block (used as the testing data), we combine the rest 4 blocks as the training data. The genes are selected with AIC differences using the training data only, and the 1-nearest neighbor classifier utilizes the class labels of training data only for predicting class labels of the testing data. To simplify the procedure, we fix the model to be either log-normal hurdle (with zero-inflation) or log-normal (without zero-inflation). We repeat the procedure so that each block serves as the testing data once. The prediction error rate is calculated based on the predictions on the 5 testing data sets.

Table 3 summarizes the result of the estimated perdition error rates based on the 5-fold cross-validation with 1-nearest neighbor classifier as described above. As the number of top selected genes increases, roughly speaking, the prediction error rate first decreases and then increases. The best prediction error rate 0 is attained at 50 genes. In other words, the best number of genes for this data is 50, which is chosen by the 5-fold cross-validation. For comparison purpose, the prediction error rate using the top 7 principal components is listed in Table 3 as well. Estimated by the 5-fold cross-validation, the prediction error rate of 7PCA is 0.0037, which is worse than the top 50 selected genes’ and also higher than its training error rate 0.02 listed in Table 2.

**Figure 6:**
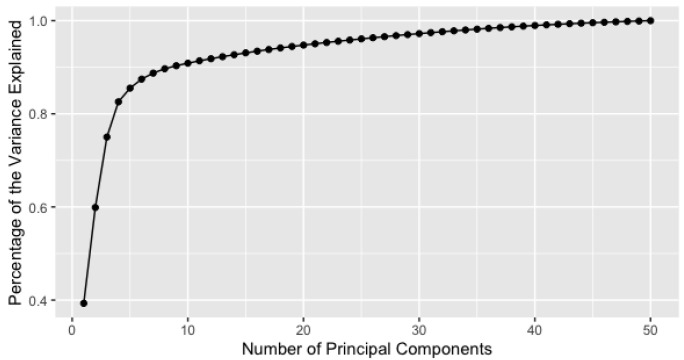
Cumulative proportions of variance explained by various numbers of principal components based on the 50 selected genes.

For comparison purpose, we further apply principal components analysis (PCA) to the top 50 selected genes. Figure 6 shows the cumulative variance explained by various numbers of principal components of the 50 genes. We recommend the top 4 principal components, which explain 83% of the variability of the data. For readers’ reference, we need the top 10 principal components to explain 90% of the variability.

**Figure 7:**
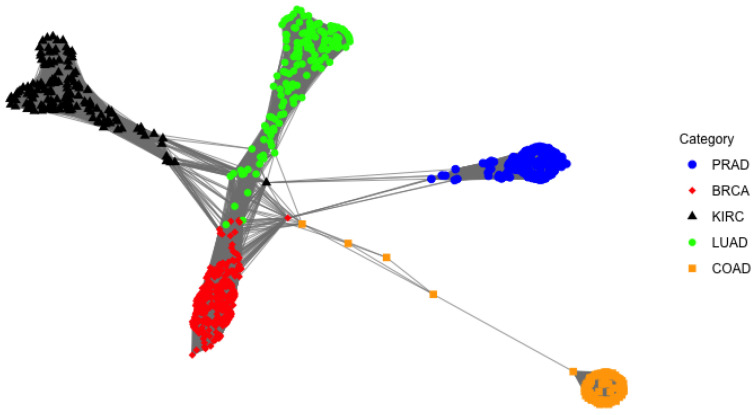
Cluster plot using TIP.

Many clustering methods have been used for gene expression data, including Ewens-Pitman Attraction (EPA), MCLUST, hierarchical clustering in conjunction with the gap statistic, *k*-means clustering in conjunction with the gap statistic, and Table Invitation Prior (TIP) [47]. Compared with other clustering methods, the TIP clustering algorithm does not require the analyst to specify the number of clusters and can still provide a good result [21]. For illustration purpose, we apply the TIP clustering method to this data with the top 4 principal components based on our top 50 selected genes. Its one-cluster plot is shown in Figure 7, where the original 5 classes seem to be well separable.

## 5. Conclusions and Discussion

In this work, we use two different real-world examples to illustrate how the sparse data model selection could be used for choosing the most significant covariates. The first example is the vaginal microbiome which is dominated by one or two species of *Lactobacillus*. The vaginal microbiome dataset is longitudinal and we aim to compare the *Lactobacillus* abundance between pregnant and non-pregnant groups over 38 time intervals (weeks). Based on our proposed variable selection method, the two groups are statistically different after week 22. In addition, we compare the estimated parameters of the two groups at each time point and show the differences in terms of model parameters. Our selected time intervals overlap with the MetaLonDA method to some extent. This work can be extended to other vaginal microbiome species as well. The second example is the gene expression data, which is high dimensional, and the subjects are categorized into 5 different cancer groups. The goal is to select the most important genes among a list of 2426 genes. Based on our variable selection procedure and PCA, we select the top 50 genes with 100% prediction accuracy. In addition, we pick up the first 4 principal components, which explain about 83% of the variability. Finally, we utilize the TIP clustering algorithm to the gene expression data with the top 4 principal components based on our top 50 selected genes, the 5 cancer classes are well separable.

## Figures and Tables

**Table 1:** KS-test *p* values for combined data (N=52) under 12 distributions at three different weeks.

Time (Week)	N	ZIN	NH	HN	ZIHN	HNH	LN	ZILN	LNH	E	ZIE	EH
week 10	1.000	0.390	1.000	0.315	0.510	0.000	0.000	0.000	0.720	0.335	0.510	0.015
week 22	1.000	0.160	1.000	0.605	0.625	0.000	0.000	0.000	0.955	0.100	0.300	0.020
week 36	1.000	0.175	1.000	0.560	0.650	0.015	0.000	0.000	0.960	0.525	0.450	0.030

Note: N: Normal; ZIN: Zero-inflated Normal; NH: Normal Hurdle; HN: Half-normal; ZIHN: Zero-inflated Half-normal; HNH: Half-normal Hurdle; LN: Log-normal; ZILN: Zero-inflated Log-normal; LNH: Log-normal Hurdle; E: Exponential; ZIE: Zero-inflated Exponential; EH: Exponential Hurdle.

**Table 2:** Training error rate by 1-nearest neighbor classifier with various number of genes or PCAs.

Number of Genes	Prediction Error
20	0.1500
50	0.0037
100	0.0037
2426	0.0012
7PCA	0.0200

**Table 3:** Estimated prediction error rate by 5-fold cross-validation with 1-nearest neighbor classifier.

Number of Genes	Prediction Error
10	0.0480
20	0.0012
30	0.0012
40	0.0024
50	0
60	0.0012
100	0.0012
7PCA	0.0037

**Figure A1:**
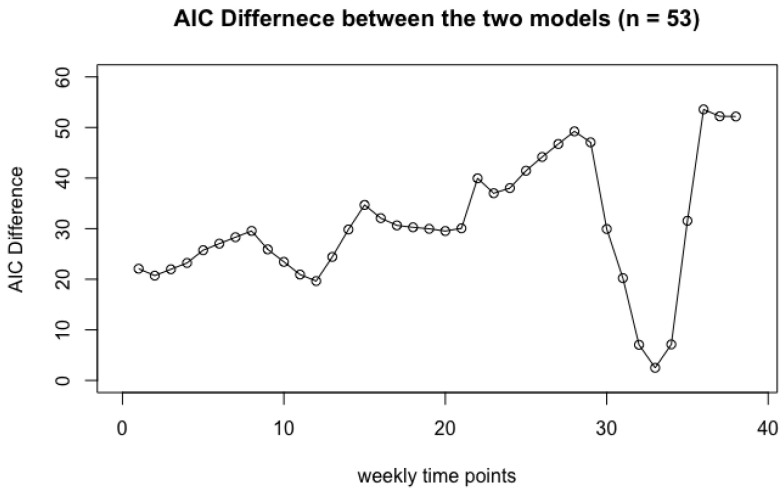
AIC difference over 38 weeks of pregnancy with the outlier.

## Data Availability

The vaginal microbiome data are available in Romero et al. (2014)’s study [2]. The gene expression data are publicly available from the UCI Machine Learning Repository (https://archive.ics.uci.edu/ml/datasets/gene+expression+cancer+RNA-Seq#, accessed on 4 November 2022) (see also Section 3).

## Supplementary Materials

The following supporting information can be downloaded at: https://www.mdpi.com/article/10.3390/genes14020403/s1, Table S1: KS-test *p* values for combined data (N=52) under 12 distributions at 38 different weeks; Table S2: KS-test *p* values for non-pregnant group (N=30) under 12 distributions at 38 different weeks; Table S3: KS-test *p* values for pregnant group (N=22) under 12 distributions at 38 different weeks; Table S4: Estimated parameters of non-pregnant and pregnant groups starting week 21.

## Appendix A. Influence of Outlier in Vaginal Microbiome Analysis

Figure A1 shows the unstable AIC differences between the two groups over time when the outlier presents (see Section 4.1). We remove the outlier from the non-pregnant samples for our analysis.

## Appendix B. More KS-Test Results for Vaginal Microbiome Analysis

**Table A1:** KS-test *p* values for non-pregnant group (N=30) under 12 distributions at different weeks.

Time (Week)	N	ZIN	NH	HN	ZIHN	HNH	LN	ZILN	LNH	E	ZIE	EH
week 10	1.000	0.395	1.000	0.460	0.580	0.020	0.000	0.000	0.870	0.330	0.725	0.040
week 22	1.000	0.480	1.000	0.520	0.420	0.010	0.000	0.000	0.785	0.450	0.590	0.025
week 36	1.000	0.160	1.000	0.480	0.790	0.020	0.000	0.000	0.955	0.145	0.375	0.015

Note: N: Normal; ZIN: Zero-inflated Normal; NH: Normal Hurdle; HN: Half-normal; ZIHN: Zero-inflated Half-normal; HNH: Half-normal Hurdle; LN: Log-normal; ZILN: Zero-inflated Log-normal; LNH: Log-normal Hurdle; E: Exponential; ZIE: Zero-inflated Exponential; EH: Exponential Hurdle.

**Table A2:** KS-test *p* values for pregnant group (N=22) under 12 distributions at different weeks.

Time (Week)	N	ZIN	NH	HN	ZIHN	HNH	LN	ZILN	LNH	E	ZIE	EH
week 10	1.000	0.355	1.000	0.505	0.385	0.170	0.000	0.000	0.390	0.500	0.130	0.130
week 22	1.000	0.250	1.000	0.295	0.490	0.415	0.020	0.000	0.000	0.135	0.005	0.170
week 36	1.000	0.380	1.000	0.635	0.975	0.560	0.005	0.000	0.000	0.425	0.550	0.410

Note: N: Normal; ZIN: Zero-inflated Normal; NH: Normal Hurdle; HN: Half-normal; ZIHN: Zero-inflated Half-normal; HNH: Half-normal Hurdle; LN: Log-normal; ZILN: Zero-inflated Log-normal; LNH: Log-normal Hurdle; E: Exponential; ZIE: Zero-inflated Exponential; EH: Exponential Hurdle.

For readers’ reference, extended versions of Table A1 and Table A2 can be found in the Supplementary Materials.

## Appendix C. List of 50 Selected Genes for Gene Expression Data

Below is the list of the 50 selected genes based on all 801 samples (see Section 4.2).

 "gene_14646" "gene_12695" "gene_17688" "gene_15945" "gene_5394"

 "gene_12209" "gene_1054" "gene_3598" "gene_7235" "gene_11440"

 "gene_4979" "gene_2288" "gene_6162" "gene_16817" "gene_15898"

 "gene_4467" "gene_3946" "gene_16392" "gene_11566" "gene_1510"

 "gene_9181" "gene_16246" "gene_16337" "gene_16169" "gene_10489"

 "gene_9680" "gene_998" "gene_9176" "gene_4833" "gene_19661"

 "gene_15447" "gene_12013" "gene_7964"  "gene_13210" "gene_3461"

 "gene_3737" "gene_15896" "gene_13497" "gene_17801" "gene_15633"

 "gene_706" "gene_10460" "gene_3862" "gene_10950" "gene_10284"

 "gene_9626" "gene_14866" "gene_3439" "gene_4618" "gene_3458"
